# Understanding the Interaction between Cyclists’ Traffic Violations and Enforcement Strategies: An Evolutionary Game-Theoretic Analysis

**DOI:** 10.3390/ijerph17228457

**Published:** 2020-11-15

**Authors:** Tianpei Tang, Yuntao Guo, Guohui Zhang, Hua Wang, Quan Shi

**Affiliations:** 1 School of Transportation and Civil Engineering, Nantong University, Nantong 226019, China; tangtianpei@ntu.edu.cn (T.T.); shi.q@ntu.edu.cn (Q.S.); 2 Department of Traffic Engineering & Key Laboratory of Road and Traffic Engineering, Ministry of Education, Tongji University, 4800 Cao’an Road, Shanghai 201804, China; 3 Civil and Environmental Engineering, University of Hawaii, Honolulu, HI 96822, USA; guohui@hawaii.edu; 4 Department of Civil and Environmental Engineering, National University of Singapore, Kent Ridge 119077, Singapore; hwang191901@gmail.com

**Keywords:** cyclists, traffic violations, enforcement strategy, evolutionary game theory, cumulative prospect theory

## Abstract

An evolutionary game-theoretic analysis method is developed in this study to understand the interactions between cyclists’ traffic violations and the enforcement strategies. The evolutionary equilibrium stabilities were analysed under a fixed (FPS) and a dynamic penalty strategy (DPS). The simulation-based numerical experiments show that: (i) the proposed method can be used to study the interactions between traffic violations and the enforcement strategies; (ii) FPS and DPS can reduce cyclists’ probability of committing traffic violations when the perceived traffic violations’ relative benefit is less than the traffic violation penalty and the enforcement cost is less than the enforcement benefit, and using DPS can yield a stable enforcement outcome for law enforcement compared to using FPS; and (iii) strategy-related (penalty amount, enforcement effectiveness, and enforcement cost) and attitudinal factors (perceived relative benefit, relative public image cost, and cyclists’ attitude towards risk) can affect the enforcement strategy’s impacts on reducing cyclists’ traffic violations.

## 1. Introduction

Due to the increasing congestion and pollution, there is an increasing demand for mobility alternatives to driving such as riding a human-powered bike or an electric bike (i.e., e-bikes equipped with bicycle pedals and e-bikes in scooter forms, hereafter referred to as “e-bike”). The ownership of e-bikes has skyrocketed over the past decade around the world. In China, 3 million e-bikes were sold and over 200 million were on the road in 2016 compared to just a few thousand in 1998 [1,2]. Between 2005 and 2012, annual sales of e-bikes in Switzerland increased from 1792 to 52,941 at an average annual growth rate of 62.2% [3]. Although the increasing usage of human-powered bikes/e-bikes offers a cheap, convenient, and environmentally friendly travel alternative, human-powered bike/e-bike-related traffic accidents also increase significantly. Traffic violation of cyclists (i.e., bike and e-bike riders) is one of the primary reasons for the increasing cyclist-related traffic accidents around the world. Traffic violation in this study is defined as the intention to disobey some traffic rules to gain some personal benefit such as reducing travel time. For example, in 2005 a survey of cyclists and pedestrians in Florida reported that nearly 15% of cyclist-related crashes were caused by cyclists’ right-of-way violations [4]. In North Carolina municipalities between 2008 and 2012, over 10% of the total cyclist-related accidents were caused by cyclists running a red light [5]. In Boston between 2011 and 2013, over 20% of cyclist-related accidents were caused by cyclists running a red light or failing to stop for a stop sign [6]. A recent study in Suzhou, China found that a majority of e-bike riders often commit traffic violations which can lead to a higher crash risk [7]. Some studies suggested that if traffic violations such as running a red light and reckless cycling can be eliminated, the total number of cyclists-related fatalities and injuries can be significantly reduced [8]. It is important for authorities to improve road safety by enforcing traffic rules and reducing cyclists’ traffic violations. In practice, traffic enforcement often relies on police officers and automatic detecting equipment (e.g., red-light cameras and speed radars) for identifying traffic rule violators [9,10,11,12,13,14,15,16]. However, it can be financially challenging and impractical to allocate resources to enforcement on every segment and intersection. Hence, extensive efforts have been spent on allocating enforcement resources to locations and subgroups of cyclists with a higher probability of committing traffic violations.

Four types of factors have been identified in the literature that affect the probability of cyclists’ traffic violations, including sociodemographic (e.g., age and gender), psychological (e.g., attitude and subjective norms), riding condition (e.g., if they are carrying a passenger and using a phone), and ambient road environment (e.g., traffic volume and intersection types) [17,18,19,20,21,22,23,24,25,26,27,28,29,30,31]. Despite that these studies provided valuable information for improving road safety, they cannot capture the interdependency between cyclists’ traffic violations and enforcement strategies (e.g., police patrol intensity and penalty severity for traffic violations). It means that, within a short time, cyclists are more likely to commit traffic violations when the enforcement is relatively relaxed. In response to the increase in traffic violations, law enforcement would impose a stricter enforcement strategy to counter that. Similar recommendations were made in the literature related to reducing drivers’ traffic violations [14,32,33,34]. 

A few studies tried to study similar interdependency between traffic violations and enforcement strategies related to drivers and law enforcement using the traditional game theory [35,36]. Bjørnskau and Elvik [35] found that adopting stricter penalties cannot influence drivers’ traffic violations, while Kim and Kim [36] suggested that drivers are less likely to commit traffic violations when law enforcement increases the penalty of traffic violations. These studies illustrate the possibility of applying a similar framework to investigate the interaction between the probability of cyclists’ traffic violations (i.e., how likely cyclists commit traffic violations) and the probability of law enforcement’s enforcing traffic rules (i.e., the intensity of police patrols or the number of traffic cameras on road segments or intersections). However, these studies assumed both drivers and law enforcement would make rational choices and cannot capture the impacts of some other factors (e.g., perceived relative benefit, enforcement cost, and relative public image cost) on the interaction between drivers and law enforcement.

This study proposes an evolutionary game theory framework to investigate the interaction between cyclists’ traffic violations and enforcement strategy. In summary, the contributions of this study are: (i) it can capture the dynamics actions and responses of cyclists and law enforcement and relax rational choices of cyclists and law enforcement with bounded rationality; (ii) it can be used to study the impacts of factors such as penalty amount, enforcement effectiveness, perceived relative benefit, enforcement cost, relative public image cost, and cyclists’ attitude towards risk (risk attitude coefficients, loss aversion coefficient, and decision weight) on the probabilities of cyclists’ traffic violations and law enforcement’s enforcing traffic rules; (iii) this proposed model framework is helpful for law enforcement to achieve a more stable reduction in probability of cyclists’ traffic violations by adopting a dynamic penalty strategy compared to a fixed penalty strategy.

## 2. An Evolutionary Game Theory-Based Framework

### 2.1. Proposed Framework

In the proposed framework, there are two parties (also known as “agents” or “players”): one is cyclists and the other one is law enforcement. Both cyclists and law enforcement are described as a collective of cyclists and law enforcement entities. Cyclists can choose to either “commit traffic violations” or “not commit traffic violations”, while law enforcement can choose to either “enforce traffic rules” or “not enforce traffic rules”. To be specific, the probability of committing traffic violations for cyclists depends on the payoffs from “commit traffic violations” and “not commit traffic violations” (e.g., travel time saving by committing traffic violations). The probability of enforcing traffic rules for law enforcement depends on the payoffs form “enforcing traffic rules” and “not enforcing traffic rules” (e.g., benefits of the collection of fines and positive public image by enforcing traffic rules). The summation of these probabilities for each party equal to one.

Table 1 illustrates the payoff matrix for the potential four outcomes based on the interactions between cyclists and law enforcement. Note that all the values in Table 1 represent the average value of the party or each enforcement/penalty, and each payoff represents the average payoff of an action. If cyclists commit traffic violations and law enforcement enforces traffic rules (Outcome 1), the payoff to cyclists equals to the *perceived relative benefit of* traffic violations (ΔV) (i.e., the perceived benefit of each violation minus not doing it) minus the average *traffic violation penalty* given by law enforcement. We assume that the cyclists’ perceived benefit of committing traffic violations will always be larger than the perceived benefit of not committing traffic violations (ΔV>0). The traffic violation penalty equal to the product of the *enforcement effectiveness* (r) (i.e., the percentage of violations that are successfully caught and enforced, 0≤r≤1) and the average *penalty amount* ($C_{p}$), $C_{p}>0$. In Outcome 1, law enforcement’s payoff equals to the traffic violation penalty minus the average *enforcement cost* ($C_{e}$), $C_{e}>0$. In Outcome 2, cyclists commit traffic violations and law enforcement does not enforce traffic rules, and cyclists’ payoff equals to ΔV, while law enforcement’s payoff equals to the negative value of the *relative public image cost* (−ΔI) (i.e., it equals to the cost of the negative public image by not punishing violations and ΔI>0). When cyclists do not commit traffic violations and law enforcement enforce traffic rules (Outcome 3), the payoff for cyclists is zero as the perceived relative benefit is a relative term. In this outcome, the payoff for law enforcement equals to the negative value of the average enforcement cost ($-C_{e}$). It is assumed that enforcing traffic rules will not generate public image benefits as enforcing traffic rules is considered as law enforcement’s responsibility. In Outcome 4, cyclists do not commit traffic violations and law enforcement does not enforce traffic rules, and the payoff for cyclists and law enforcement are both zero. 

It is important to note that the payoffs to cyclists and law enforcement are simplified for illustration purposes. Most of the assumptions related to payoffs can be relaxed. The main objective of this study is to analyze the evolutionary stable strategies (ESSs) and their conditions in the game regardless of the exact utility (e.g., income or cost) as long as the nature of utility meets the rationality constraints as required in a real traffic scenario. 

Given the payoffs in Table 1, the models for violations and enforcement decisions follow Expected Utility Theory [37,38,39], whereby one party’s decision utility depends upon the expected action of the other party. Therefore, payoff functions of two parties are inter-related. The expected utility decision models are shown in Equations (1)–(6).

The probabilities of the co-decision utilities are the anticipations or beliefs of the other party’s decisions, p and q (p∈[0,1] and q∈[0,1]). p is law enforcement’s estimated probability that cyclists will commit traffic violations in the period. q is the cyclists’ estimated probability that law enforcement will enforce traffic rules which represents the monitoring intensity of law enforcement such as police patrols and traffic cameras.

The expected utility decision models of committing (Equation (1)) and not committing traffic violations (Equation (2)), and the average expected utility decision model for cyclists (Equation (3)), can be calculated as follows:

(1) $$U_{v1}=q\left(\Delta V-rC_{p}\right)+\left(1-q\right)\Delta V=\Delta V-qrC_{p}$$

(2) $$U_{v2}=q\left(0\right)+\left(1-q\right)\left(0\right)=0$$

(3) $${\bar{U}}_{v}=pU_{v1}+\left(1-p\right)U_{v2}=p\left(\Delta V-qrC_{p}\right)$$

where $U_{v1}$: Expected utility for committing traffic violations of cyclists, $U_{v2}$: Expected utility for not committing traffic violations of cyclists and ${\bar{U}}_{v}$: Average expected utility for cyclists’ decision.

The expected utility decision models of enforcing (Equation (4)) and not enforcing traffic rules (Equation (5)), and the average expected utility decision model for law enforcement ((Equation (6)), can be calculated as follows:

(4) $$U_{e1}=p\left(rC_{p}-C_{e}\right)+\left(1-p\right)\left(-C_{e}\right)=prC_{p}-C_{e}$$

(5) $$U_{e2}=p\left(-\Delta I\right)+\left(1-p\right)\left(0\right)=-p\Delta I$$

(6) $${\bar{U}}_{e}=qU_{e1}+\left(1-q\right)U_{e2}=q\left(prC_{p}-C_{e}\right)-p\Delta I\left(1-q\right)$$

where $U_{e1}$: Expected utility for enforcing traffic rules of law enforcement, $U_{e2}$: Expected utility for not enforcing traffic rules of law enforcement and ${\bar{U}}_{e}$: Average expected utility for law enforcement’ decision.

Then, the replicator equation is used to capture the evolutionary process through selection dynamics [40,41]. It can be used to measure the changes to a party’s probability of choosing a strategy throughout different time periods. The replicator equation of cyclists’ decision is denoted as $f\left(p\right)=\frac{dp}{dt}$, and the replicator equation of law enforcement’s decision is denoted as $f\left(q\right)=\frac{dq}{dt}$ [42]. The replicator equations can be written as:

(7) $$f\left(p\right)=\frac{dp}{dt}=p\left(U_{v1}-{\bar{U}}_{v}\right)=p\left(1-p\right)\left(\Delta V-qrC_{p}\right)$$

(8) $$f\left(q\right)=\frac{dq}{dt}=q\left(U_{e1}-{\bar{U}}_{e}\right)=q\left(1-q\right)\left(prC_{p}+p\Delta I-C_{e}\right)$$

In Equations (7) and (8), the penalty amount ($C_{p}$) can be flexible. It can be a fixed penalty strategy (FPS) or a dynamic penalty strategy (DPS). Under FPS, the penalty amount during the study period is fixed, while under DPS, the penalty amount changes at the end of every time unit (one day or several days) within the study period. The evolutionary equilibrium stabilities under these two penalty strategies are discussed in details in Section 2.2 and Section 2.3.

The perceived relative benefit (ΔV) can be influenced by cyclists’ attitude towards risk. To capture the impacts of these attitude-related factors on the perceived relative benefit of traffic violations, Cumulative Prospect Theory (CPT) is used [43,44]. CPT is used to model descriptive decisions under risk and uncertainty. Based on CPT, people tend to measure the perceived benefits/costs of the possible outcomes to a reference point (most of the time is the status quo) rather than the outcome’s absolute value. People can have various risk attitudes towards gains (i.e., outcomes above the reference point) and losses (i.e., outcomes below the reference point). Most people are risk-averse. It means that most people value more about the potential losses than the same amount of potential gains when exposed to uncertainty [45,46]. The cyclists’ perceived relative benefit is formulated as follows:

(9) $$\Delta V=\overset{2}{\underset{i=1}{\sum }}\omega \left(p_{i}\right)v\left(x_{i}\right)$$

where,$\;\omega \left(p_{i}\right)$ is cyclists’ decision weight (i.e., how much weight they assigned to each risky outcome’s probability) on their subjective perception towards the probabilities of two risky outcomes ($p_{i}$), *i* = 1 or 2, $p_{1}$ refers to the probability of not getting punished for traffic violations, and $p_{2}$ refers to the probability of getting punished for traffic violations. $\omega \left(p_{1}\right)+\omega \left(p_{2}\right)<1$ is based on Kahneman and Tversky [43]. $v\left(x_{i}\right)$ is cyclists’ value function for the benefit of committing traffic violation with/without being punished compared to the reference point, where $x_{1}$ refers to the difference between the benefit received (i.e., benefit of committing traffic violations without being punished) and the reference point, and $x_{2}$ refers to the difference between the benefit received (i.e., benefit of committing traffic violations but got punished) and the reference point. The reference point is the benefit of not committing traffic violations.

The value function proposed by Tversky and Kahneman [44] is applied in this study. It can be written as follows:

(10) $$v\left(x_{i}\right)=\{\begin{array}{cc} x_{i}{}^{\alpha }, & x_{i}\geq 0\\ -\lambda {\left(-x_{i}\right)}^{\beta }, & x_{i}<0 \end{array}$$

where, α, β are the risk attitude coefficients that determine the convexity or concavity of the value function shape, and α, β∈[0,1]. These two coefficients are related to cyclists’ risk attitude towards traffic violations. The smaller the risk attitude coefficients, the higher the risk that cyclists perceive the violations. λ is the loss aversion coefficient that can capture cyclists’ sensitivity to possible losses when punished for committing traffic violations, and λ≥1.

By combining Equations (9) and (10), ΔV can be written as follows:

(11) $$\Delta V=\omega \left(p_{1}\right)v\left(E-0\right)+\omega \left(p_{2}\right)v\left(E-C_{p}-0\right)=\omega \left(p_{1}\right)v\left(E\right)+\omega \left(p_{2}\right)v\left(E-C_{p}\right)$$

where, E is the benefit received from each violation for cyclists. 

Assuming $C_{p}>E$, the final conversion equation of ΔV is represented as follows:

(12) $$\Delta V=\omega \left(p_{1}\right)E^{\alpha }-\omega \left(p_{2}\right)\lambda {\left(C_{p}-E\right)}^{\beta }$$

### 2.2. Evolutionary Equilibrium Stability under FPS

Under FPS, the fixed penalty amount is calculated as follows:

(13) $$C_{p}=C_{p\left(f\right)}$$

By substituting Equation (13) into Equations (7) and (8), under FPS, the replicator equations can be written as:

(14) $$f\left(p\right)=\frac{dp}{dt}=p\left(1-p\right)\left(\Delta V-qrC_{p\left(f\right)}\right)$$

(15) $$f\left(q\right)=\frac{dq}{dt}=q\left(1-q\right)\left(prC_{p\left(f\right)}+p\Delta I-C_{e}\right)$$

In an evolutionary game model, the trajectory emitted from an arbitrarily small neighborhood evolves towards a certain asymptotically stable balance point, which is called ESS [47]. If a sufficient probability of parties adopts a certain strategy that achieves ESSs, then the system will remain stable. In Equations (14) and (15), the transformation rate should be zero based on the definitions of ESSs, i.e., $f\left(p\right)=\frac{dp}{dt}=0$ and $f\left(q\right)=\frac{dq}{dt}=0$. Thereby, the potential five ESSs are: $E_{1}=\left(0,0\right)$, $\;E_{2}=\left(0,1\right)$, $\;E_{3}=\left(1,0\right)$, $\;E_{4}=\left(1,1\right)$, $E_{5}=\left(p^{*},q^{*}\right)$, where $p^{*}=\frac{C_{e}}{rC_{p\left(f\right)}+\Delta I}$, $q^{*}=\frac{\Delta V}{rC_{p\left(f\right)}}$.

Friedman’s [48] study provided the ESSs condition for the evolutionary game. Specifically, for an equilibrium state to be asymptotically stable, the determinant of the Jacobian matrix J should be positive (det J>0) and the trace of Jacobian matrix J should be negative (tr J<0). Any state meets the above condition is an ESS. By using the replicator Equations of (14) and (15), the Jacobian matrix J can be written as:

(16) $$J=\left[\begin{array}{cc} \frac{\partial f\left(p\right)}{\partial p} & \frac{\partial f\left(p\right)}{\partial q}\\ \frac{\partial f\left(q\right)}{\partial p} & \frac{\partial f\left(q\right)}{\partial q} \end{array}\right]=\left[\begin{array}{cc} \left(1-2p\right)\left(\Delta V-qrC_{p\left(f\right)}\right) & \left(p^{2}-p\right)rC_{p\left(f\right)}\\ \left(q-q^{2}\right)(rC_{p\left(f\right)}+\Delta I) & \left(1-2q\right)\left(prC_{p\left(f\right)}+p\Delta I-C_{e}\right) \end{array}\right]$$

Then, the det J and tr J can be given by:

(17) $$det\;J=\left(1-2p\right)\left(\Delta V-qrC_{p\left(f\right)}\right)\left(1-2q\right)\left(prC_{p\left(f\right)}+p\Delta I-C_{e}\right)-\left(q-q^{2}\right)(rC_{p\left(f\right)}+\Delta I)\left(p^{2}-p\right)rC_{p\left(f\right)}$$

(18) $$tr\;J=\left(1-2p\right)\left(\Delta V-qrC_{p\left(f\right)}\right)+\left(1-2q\right)\left(prC_{p\left(f\right)}+p\Delta I-C_{e}\right)$$

Based on Equations (17) and (18), ESSs are conditioned upon the values of the following parameters: ΔV, $C_{p\left(f\right)}$, r, $C_{e}$, and ΔI, where ΔV are determined by cyclists, $C_{p\left(f\right)}$, r, and $C_{e}$ are determined by law enforcement, ΔI are determined by the public. Under FPS, we discuss all possible conditional constraints for the equilibrium stability analysis. Table 2 summarizes the determinants and traces of the Jacobian matrix J for five potential ESSs. The local stability of equilibriums for three situations are shown in Table 3, Table 4 and Table 5. Situation 1: if $\Delta V>rC_{p\left(f\right)}$ and $rC_{p\left(f\right)}+\Delta I>C_{e}$, $E_{4}=\left(1,1\right)$ is an ESS, which corresponds to a pure strategy (i.e., one party can only adopt one strategy at one time) in which cyclists commit traffic violations, and law enforcement enforces traffic rules. Situation 2: if ΔV>0 and $rC_{p\left(f\right)}+\Delta I<C_{e}$, $E_{3}=\left(1,0\right)$ is an ESS, which corresponds to a pure strategy in which cyclists commit traffic violations, and law enforcement does not enforce traffic rules. Situation 3: if $0<\Delta V<rC_{p\left(f\right)}$ and $rC_{p\left(f\right)}+\Delta I>C_{e}$, $E_{5}=\left(p^{*},q^{*}\right)$ is an unstable center, which corresponds to a mixed strategy (i.e., one party has a probability of adopting each strategy). It means that the strategy probabilities of cyclists and law enforcement will fluctuate around $\left(p^{*},q^{*}\right)$ and cannot converge. The other situations (i.e., $\Delta V=rC_{p\left(f\right)}$ or $rC_{p\left(f\right)}+\Delta I=C_{e}$) are less likely to occur [49].

### 2.3. Evolutionary Equilibrium Stability under DPS

The volatility and repeated traffic violations (i.e., in Situation 3 of FPS, the probability of committing traffic violations is constantly fluctuating) may cause law enforcement to make overly optimistic/pessimistic estimations. For example, law enforcement may introduce a penalty which is sufficient to reduce traffic violations based on an optimistic estimation under FPS. This means that an ideal penalty strategy should not only be able to reduce the total number of traffic violations but also allow law enforcement to correctly assess/predict the effectiveness of its strategies (i.e., a stable equilibrium solution). Hence, the potential of using DPS in which the penalty amount is correlated with the probability of committing traffic violations is proposed and studied. The dynamic penalty amount is calculated as follows:

(19) $$C_{p}=kC_{p\left(d\right)}$$

where k is the dynamic penalty coefficient relative to p. In this study, we first select one form of k, i.e., k=1+p, k∈[1,2] for demonstration purpose. It means the minimum penalty amount for traffic violations is $C_{p\left(d\right)}$ and the maximum penalty amount is $2C_{p\left(d\right)}$, as the probability of committing traffic violations reaches to 1.

By substituting Equation (19) into Equations (7) and (8), under DPS, the replicator equations can be given as:

(20) $$f\left(p\right)=p\left(1-p\right)\left(\Delta V-qrC_{p\left(d\right)}\left(1+p\right)\right)$$

(21) $$f\left(q\right)=q\left(1-q\right)\left(prC_{p\left(d\right)}\left(1+p\right)+p\Delta I-C_{e}\right)$$

Similarly, the candidates of five ESSs are: $E_{1}=\left(0,0\right)$, $\;E_{2}=\left(0,1\right)$, $\;E_{3}=\left(1,0\right)$, $\;E_{4}=\left(1,1\right)$, $E_{5}=\left(p^{*},q^{*}\right)$, where $p^{*}=\frac{\sqrt{{\left(rC_{p\left(d\right)}+\Delta I\right)}^{2}+4rC_{p\left(d\right)}C_{e}}-rC_{p\left(d\right)}-\Delta I}{2rC_{p\left(d\right)}}$, $q^{*}=\frac{2\Delta V}{\sqrt{{\left(rC_{p\left(d\right)}+\Delta I\right)}^{2}+4rC_{p\left(d\right)}C_{e}}+rC_{p\left(d\right)}-\Delta I}$.

Similarly, by using the replicator Equations of (20) and (21), the Jacobian matrix J is given by

(22) $$J=\left[\begin{array}{cc} qrC_{p\left(d\right)}\left(3p^{2}-1\right)+\Delta V\left(1-2p\right) & \left(p^{3}-p\right)rC_{p\left(d\right)}\\ 2pqrC_{p\left(d\right)}\left(1-q\right)-\left(q^{2}-q\right)(rC_{p\left(d\right)}+\Delta I) & \left(1-2q\right)\left(p^{2}rC_{p\left(d\right)}+p\left(rC_{p\left(d\right)}+\Delta I\right)-C_{e}\right) \end{array}\right]$$

Then, the det J and tr J can be given by

(23) $$\begin{array}{c} det\;J=\left(qrC_{p\left(d\right)}\left(3p^{2}-1\right)+\Delta V\left(1-2p\right)\right)\left(1-2q\right)\left(p^{2}rC_{p\left(d\right)}+p\left(rC_{p\left(d\right)}+\Delta I\right)-C_{e}\right)\\ -\left(2pqrC_{p\left(d\right)}\left(1-q\right)-\left(q^{2}-q\right)(rC_{p\left(d\right)}+\Delta I)\right)\left(p^{3}-p\right)rC_{p\left(d\right)} \end{array}$$

(24) $$tr\;J=qrC_{p\left(d\right)}\left(3p^{2}-1\right)+\Delta V\left(1-2p\right)+\left(1-2q\right)\left(p^{2}rC_{p\left(d\right)}+p\left(rC_{p\left(d\right)}+\Delta I\right)-C_{e}\right)$$

Under DPS, we discuss all possible conditional constraints for the equilibrium stability analysis. Table 6 summarizes the determinants and traces of the Jacobian matrix J for five potential ESSs. The local stability of equilibriums for three situations are shown in Table 3, Table 4 and Table 5. Situation 1: if $\Delta V>2rC_{p\left(d\right)}$ and $2rC_{p\left(d\right)}+\Delta I>C_{e}$, $E_{4}=\left(1,1\right)$ is an ESS. Situation 2: if ΔV>0 and $2rC_{p\left(d\right)}+\Delta I<C_{e}$, $E_{3}=\left(1,0\right)$ is an ESS. Situation 3: if $0<\Delta V<2rC_{p\left(d\right)}$ and $2rC_{p\left(d\right)}+\Delta I>C_{e}$, $\;E_{5}=\left(p^{*},q^{*}\right)$ is an ESS, which corresponds to a mixed strategy that cyclists’ strategy probability will converge to $p^{*}$ and law enforcement’s strategy probability will converge to $q^{*}$. The other situations (i.e., $\Delta V=2rC_{p\left(d\right)}$ or $2rC_{p\left(d\right)}+\Delta I=C_{e}$) are less likely to occur [49].

To evaluate the performance of the proposed framework, numerical experiments are conducted. In addition, the impacts of the penalty amount, enforcement effectiveness, perceived relative benefit, enforcement cost, relative public image cost, and cyclists’ attitude towards risk (risk attitude coefficients, loss aversion coefficient, and decision weight) on the interactions between cyclists and law enforcement are discussed. Runge-Kutta algorithm [50,51] is used to solve the proposed framework. Since the pure strategies are (1,1), (1,0), or (0,0), and the mixed strategies are $\left(\frac{C_{e}}{rC_{p\left(f\right)}+\Delta I},\frac{\Delta V}{rC_{p\left(f\right)}}\right)$ or $\left(\frac{\sqrt{{\left(rC_{p\left(d\right)}+\Delta I\right)}^{2}+4rC_{p\left(d\right)}C_{e}}-rC_{p\left(d\right)}-\Delta I}{2rC_{p\left(d\right)}},\frac{2\Delta V}{\sqrt{{\left(rC_{p\left(d\right)}+\Delta I\right)}^{2}+4rC_{p\left(d\right)}C_{e}}+rC_{p\left(d\right)}-\Delta I}\right)$, the initial strategy probabilities have no effect on the equilibrium solutions. The initial strategy probabilities are set as $\left(p_{0},q_{0}\right)=\left(0.5,0.5\right)$.

MATLAB R2014b is used to conduct numerical experiments. The values of each factor in the proposed models are shown in Table 7. In this study, t represents the smallest time unit (one day or a few days) within the study period. We assume that the cyclists or law enforcement will evaluate their behavior at the end of each time unit and determine their action for the next time unit. Their behavior will not change within the time unit. For simplicity, we define a time unit as one day. The results of numerical experiments are presented in Section 3 and Section 4.

## 3. Numerical Experiments and Discussion in Situations 1 and 2

**Figure A1:**
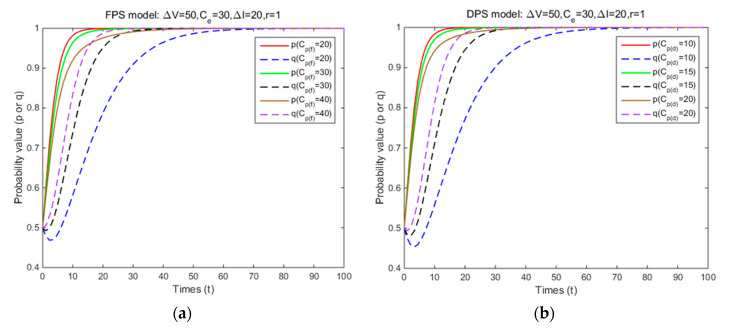
The effects of penalty amount on strategy probabilities of cyclists and law enforcement under FPS (**a**) and DPS (**b**) (Situation 1).

**Figure A2:**
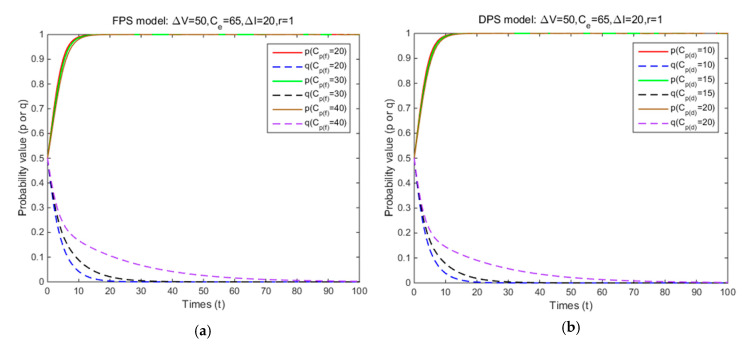
The effects of penalty amount on strategy probabilities of cyclists and law enforcement under FPS (**a**) and DPS (**b**) (Situation 2).

**Figure A3:**
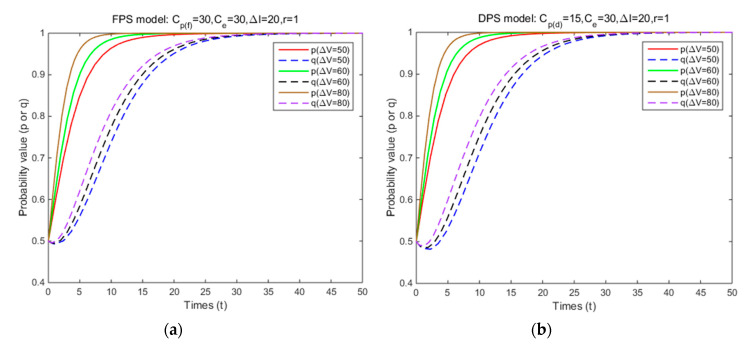
The effects of perceived relative benefit on strategy probabilities of cyclists and law enforcement under FPS (**a**) and DPS (**b**) (Situation 1).

**Figure A4:**
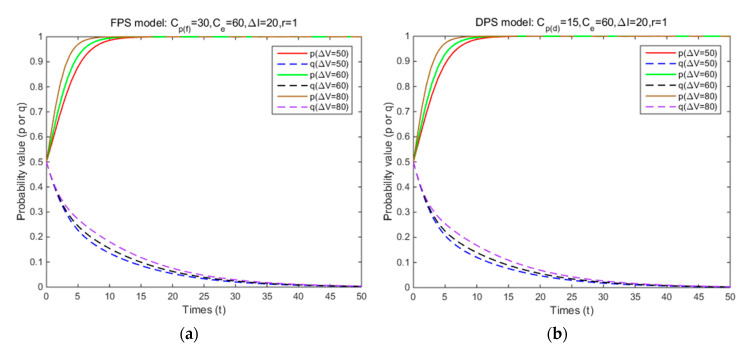
The effects of perceived relative benefit on strategy probabilities of cyclists and law enforcement under FPS (**a**) and DPS (**b**) (Situation 2).

**Figure A5:**
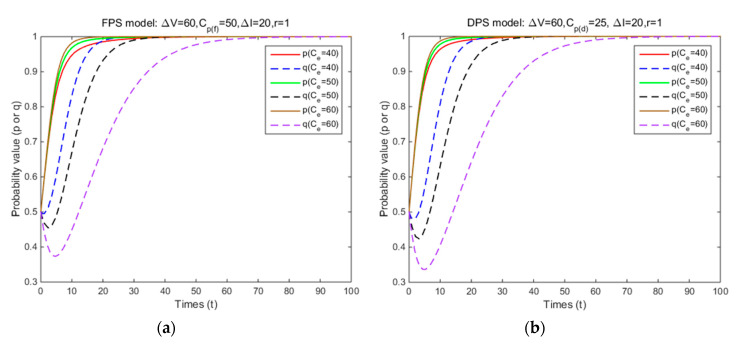
The effects of enforcement cost on strategy probabilities of cyclists and law enforcement under FPS (**a**) and DPS (**b**) (Situation 1).

**Figure A6:**
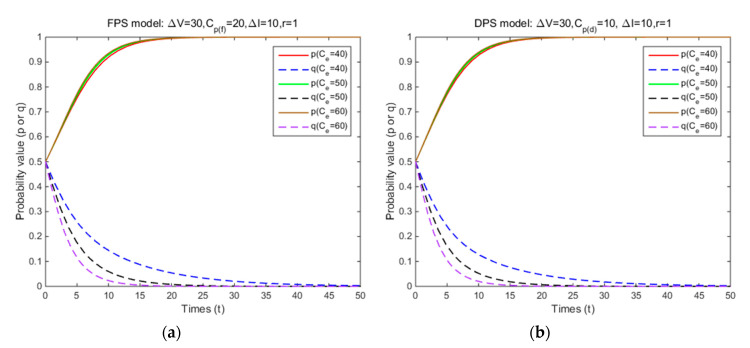
The effects of enforcement cost on strategy probabilities of cyclists and law enforcement under FPS (**a**) and DPS (**b**) (Situation 2).

**Figure A7:**
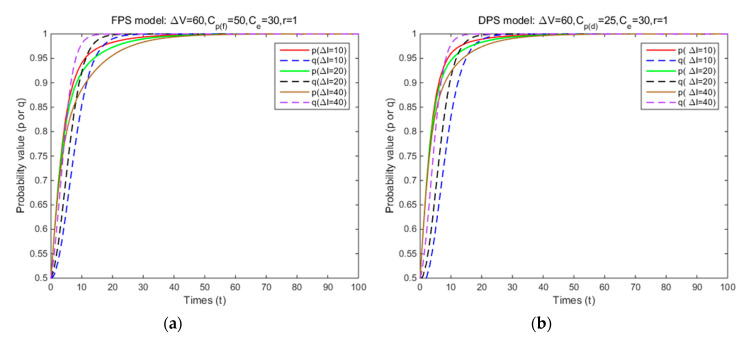
The effects of relative public image cost on strategy probabilities of cyclists and law enforcement under FPS (**a**) and DPS (**b**) (Situation 1).

**Figure A8:**
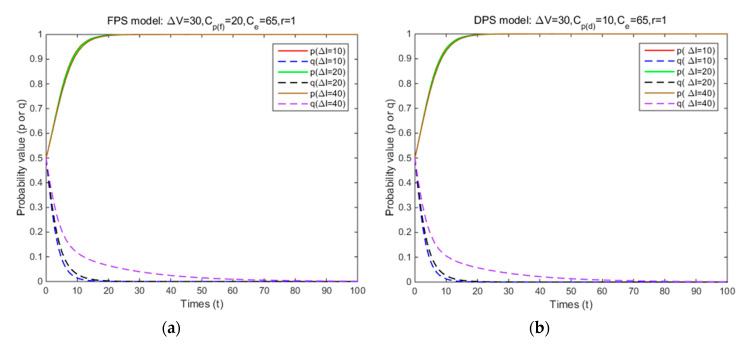
The effects of relative public image cost on strategy probabilities of cyclists and law enforcement under FPS (**a**) and DPS (**b**) (Situation 2).

In Situations 1 and 2 under FPS or DPS, the strategy choices of both cyclists and law enforcement are pure strategies. The simulation results are shown in Appendix A (Figure A1, Figure A2, Figure A3, Figure A4, Figure A5, Figure A6, Figure A7 and Figure A8). As shown in Appendix A (Figure A1, Figure A3, Figure A5 and Figure A7), if the perceived relative benefit of committing traffic violations is larger than the traffic violation penalty, and the enforcement cost is smaller than the enforcement benefit (the sum of the traffic violation penalty and the relative public image cost), the strategy probabilities of both parties converge to (1,1). This means that cyclists will commit traffic violations, and law enforcement will enforce them. If the perceived relative benefit of committing traffic violations is larger than zero and the enforcement cost is larger than the enforcement benefit, the strategy probabilities of both parties converge to (1,0) (Appendix A (Figure A2, Figure A4, Figure A6 and Figure A8)). It means that cyclists will commit traffic violations, while law enforcement will not enforce them. 

Table 8 summarizes the converging speed (i.e., the time takes to reach equilibrium solutions) changes of the cyclists’ probability of committing traffic violations and law enforcement’s probability of enforcing traffic rules in Situations 1 and 2. The benefit of committing traffic violations for cyclists increases when the penalty amount or the relative public image cost decreases, or the perceived relative benefit or the enforcement cost increases, and vice versa. The benefit of enforcing traffic rules for law enforcement increases when the penalty amount or the relative public image cost or the perceived relative benefit increases, or the enforcement cost decreases, and vice versa. These results suggest that (i) in Situations 1 and 2, cyclists needs less time (i.e., a faster converging speed) to reach the conclusion of committing traffic violations when its benefits increase and vice versa; and (ii) in Situation 1, law enforcement needs less time to reach the conclusion of enforcing traffic rules when its benefit increases and vice versa, while in Situation 2, law enforcement needs more time to reach the conclusion of not enforcing traffic rules when its benefit increases and vice versa. These results show that the increasing benefit of committing traffic violations can incentivize cyclists to commit traffic violations. For law enforcement, when the increasing benefit of enforcing traffic rules can cover the cost of enforcing traffic rules, it can incentivize law enforcement to enforce them. When the increasing benefit is not sufficient to cover the cost of enforcing traffic rules, law enforcement would choose not to enforce them. 

In Situation 1, the converging speed for cyclists under DPS is much faster compared to it under FPS, while the converging speed for law enforcement under DPS is relatively slower compared to it under FPS. In Situation 2, the converging speed for both cyclists and law enforcement is faster under DPS compared to it under FPS. Overall, the differences among the converging speeds of FPS and DPS in Situations 1 and 2 are small (i.e., less than 10% in all cases). For example, as shown in Appendix A (Figure A1), it takes about 73 days for the probability of committing traffic violations to 1 under FPS (when $C_{p\left(f\right)}=40$), while it takes around 67 days for the probability of committing traffic violations to 1 under DPS (when $C_{p\left(d\right)}=20$) in Situation 1 (Table 8). The main reason for such differences is that the penalty amount under DPS increases gradually compared to a fixed penalty under FPS. This means cyclists benefit more from committing traffic violations and law enforcement benefits less from enforcing traffic rules under DPS compared to those under FPS. That is why it takes a shorter time for cyclists to reach a final decision (Situations 1 and 2), and it takes a longer time for law enforcement to reach a final decision in Situation 1, but a shorter time in Situation 2. 

To sum up, both FPS and DPS cannot effectively reduce cyclists’ traffic violations as long as the traffic violation penalty is lower than the perceived relative benefit or the enforcement cost is higher than the benefit of enforcing traffic rules.

## 4. Numerical Experiments and Discussion in Situation 3

**Figure 1:**
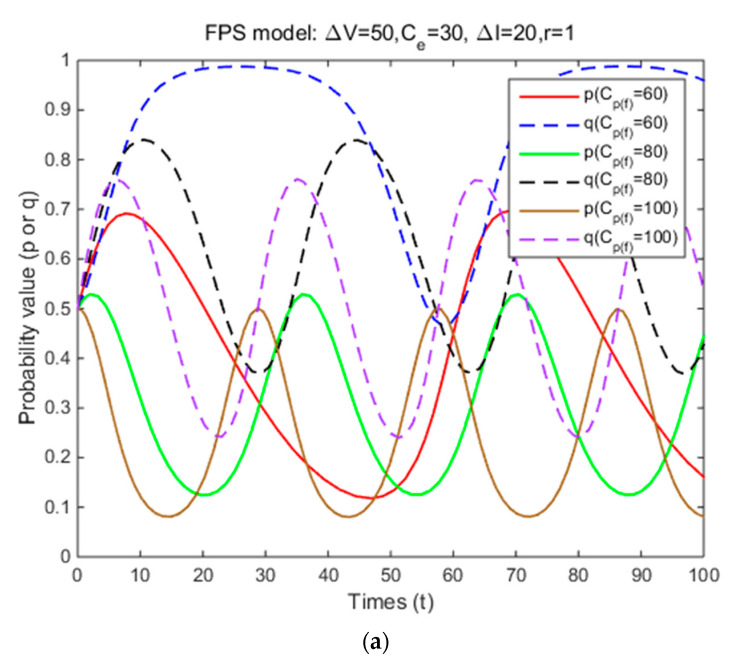

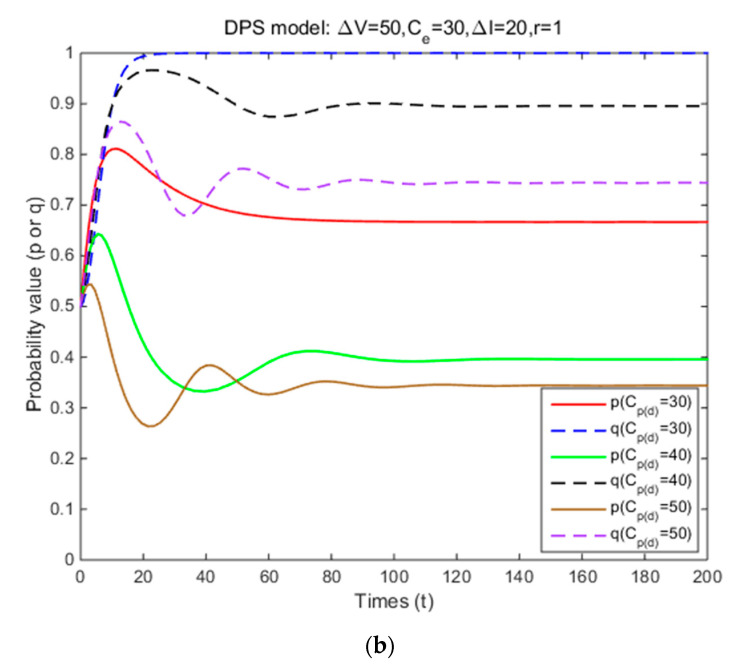
The effects of penalty amount on strategy probabilities of cyclists and law enforcement under FPS (**a**) and DPS (**b**) (Situation 3).

**Figure 2:**
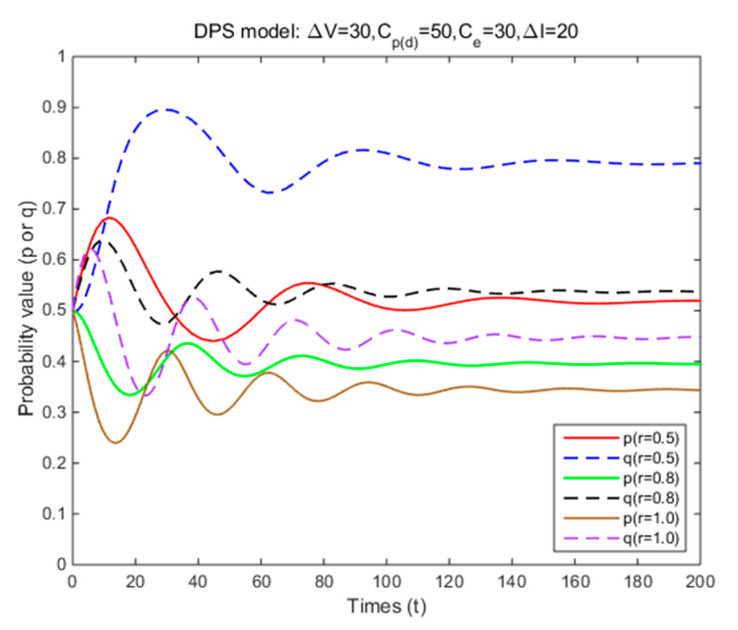
The effects of enforcement effectiveness on strategy probabilities of cyclists and law enforcement under DPS (Situation 3).

**Figure 3:**
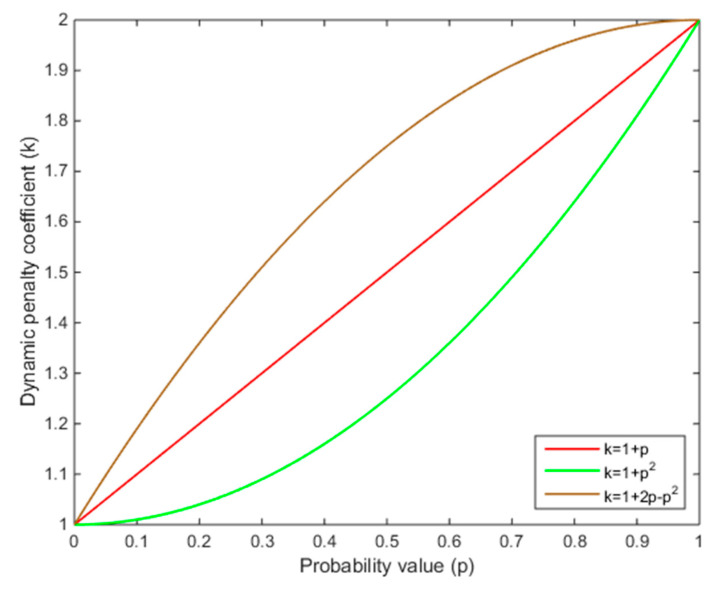
Relationship between the probability of committing traffic violations and the dynamic penalty coefficient.

**Figure 4:**
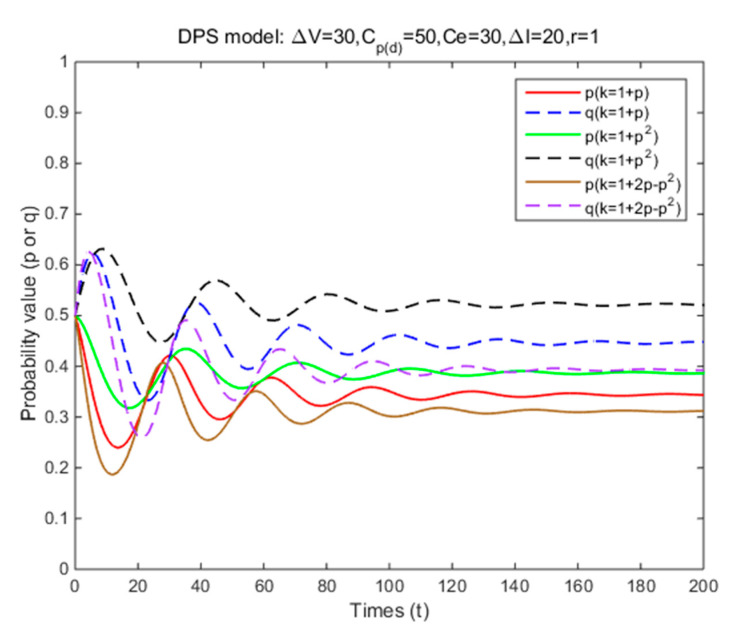
The effects of dynamic penalty coefficient on strategy probabilities of cyclists and law enforcement under DPS (Situation 3).

**Figure 5:**
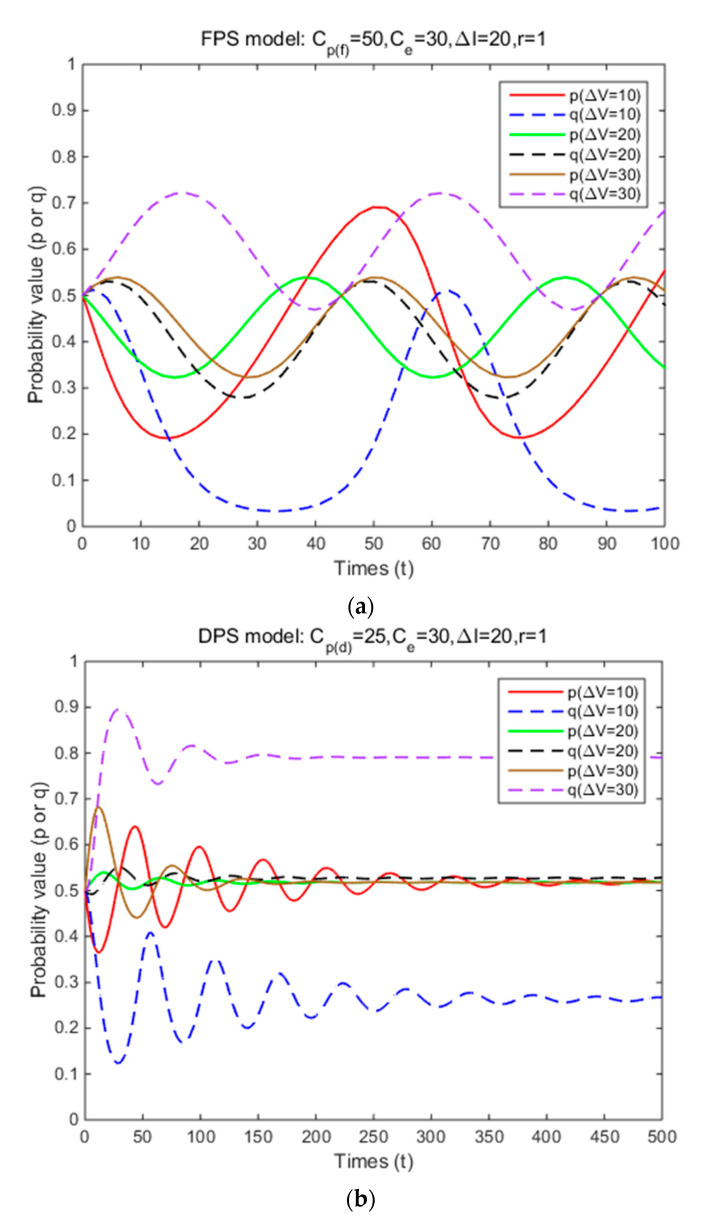
The effects of perceived relative benefit on strategy probabilities of cyclists and law enforcement under FPS (**a**) and DPS (**b**) (Situation 3).

**Figure 6:**
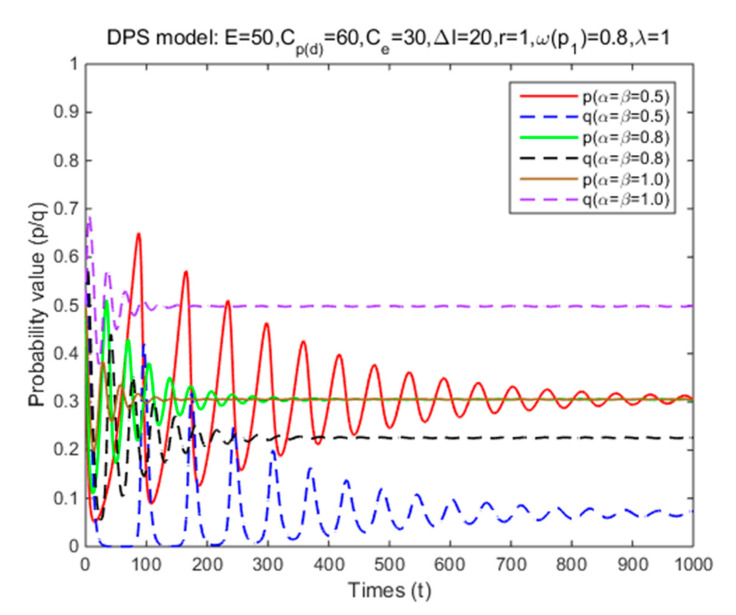
The effects of risk attitude coefficients on strategy probabilities of cyclists and law enforcement under DPS (Situation 3).

**Figure 7:**
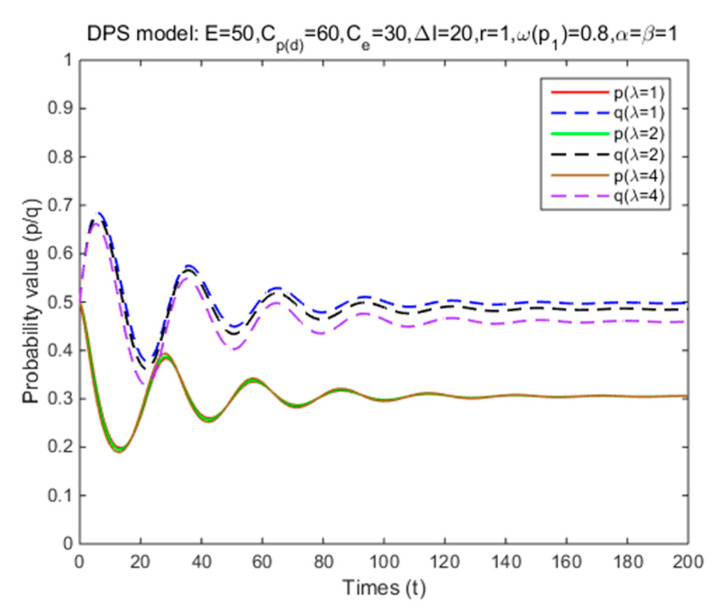
The effects of loss aversion coefficient on strategy probabilities of cyclists and law enforcement under DPS (Situation 3).

**Figure 8:**
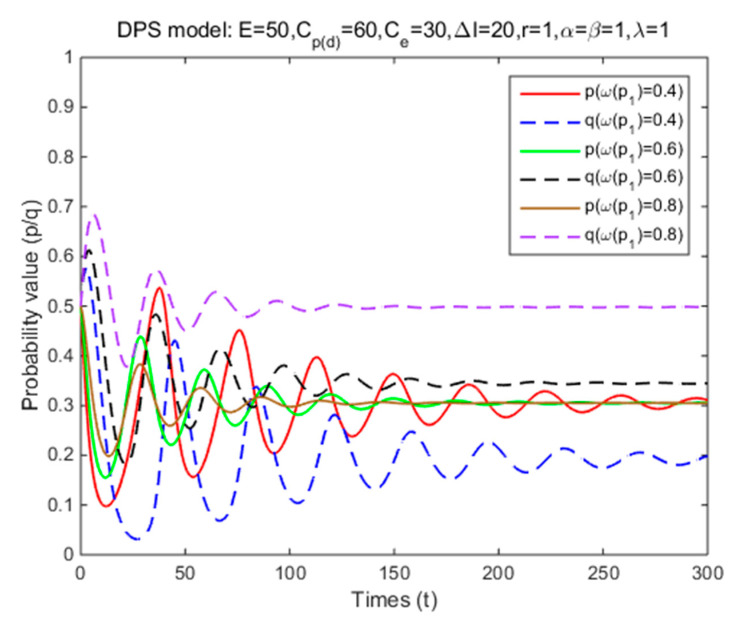
The effects of decision weight on strategy probabilities of cyclists and law enforcement under DPS (Situation 3).

**Figure 9:**
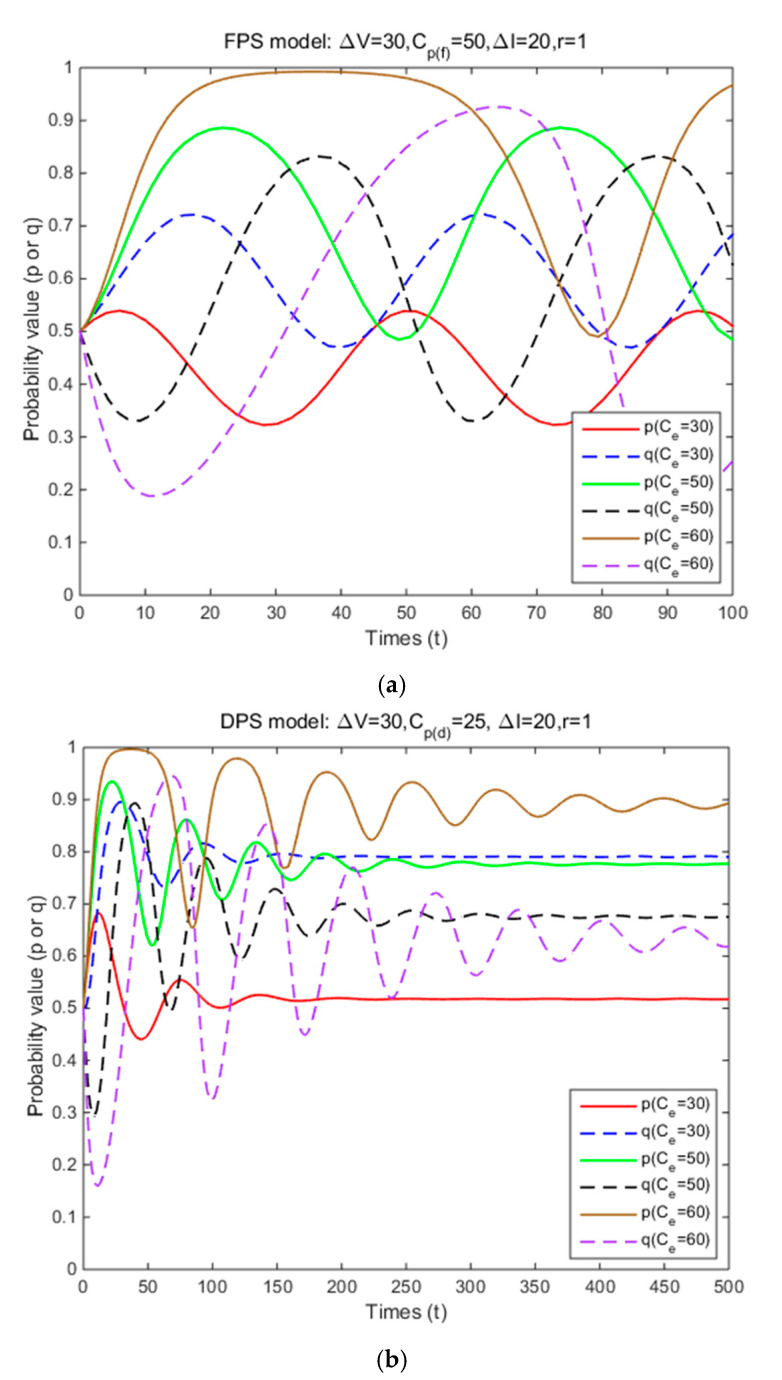
The effects of enforcement cost on strategy probabilities of cyclists and law enforcement under FPS (**a**) and DPS (**b**) (Situation 3).

**Figure 10:**
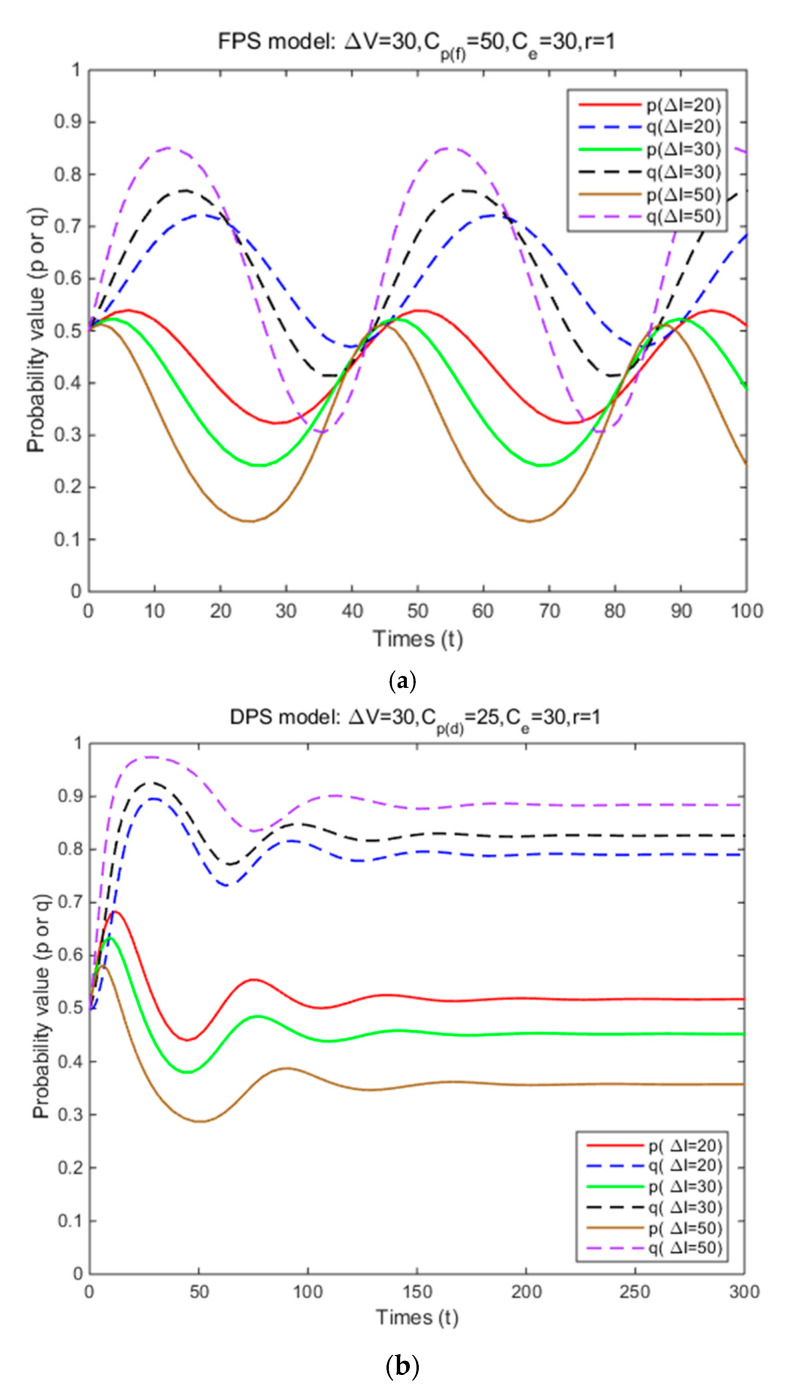
The effects of relative public image cost on strategy probabilities of cyclists and law enforcement under FPS (**a**) and DPS (**b**) (Situation 3).

In Situation 3, the perceived relative benefit of committing traffic violations is less than the traffic violation penalty, and the enforcement cost is smaller than the enforcement benefit. Under FPS or DPS, the strategy choices of both cyclists and law enforcement are mixed strategies. The first-order partial derivatives of $p^{*}$, $q^{*}$ and ΔV were conducted to illustrate the impacts of various factors on the probabilities of traffic violations and enforcing traffic rules and numerical experiment results are shown in Figure 1, Figure 2, Figure 3, Figure 4, Figure 5, Figure 6, Figure 7, Figure 8, Figure 9 and Figure 10 to further illustrate these impacts. As shown in Figure 1a, Figure 5a, Figure 9a and Figure 10a, under FPS, the strategy choice path fluctuates. This can potentially result in uncertainties in the expected outcome of the enforcement. As shown in Figure 1b, Figure 2, Figure 4, Figure 5b, Figure 6, Figure 7, Figure 8, Figure 9b and Figure 10b, under DPS, a stable expected outcome of the enforcement can be achieved. Table 9 summarizes the changes in probabilities and stabilities of traffic violations and enforcing traffic rules in Situation 3.

### 4.1. Analyzing Factors Affecting Cyclists and Law Enforcement Behavior under DPS

We solve the first-order partial derivatives of $p^{*}$ and $q^{*}$ with respect to $C_{p\left(d\right)}$, r, ΔV, $C_{e}$, and ΔI, as well as the first-order partial derivatives of ΔV with respect to α/β,  λ, and $\omega \left(p_{1}\right)$. The results are shown in Appendix B. From Equations (A1) to (A9), when the traffic violation penalty is larger than the perceived relative benefit and the enforcement cost is smaller than the enforcement benefit, if $C_{p\left(d\right)}$, r, and ΔI increase or $C_{e}$ decreases, the probability of committing traffic violations will decrease; if $C_{p\left(d\right)}$, r, and $C_{e}$ increase or ΔV and ΔI decreases, the probability of enforcing traffic rules will decrease. From Equations (A10) to (A12), when the penalty amount is more than the benefit received from each violation for cyclists, if λ increases or α/β and $\omega \left(p_{1}\right)$ decrease, the perceived relative benefit will decrease, resulting in a reduction in the probability of enforcing traffic rules.

### 4.2. The Effects of Traffic Violation Penalty

The total traffic violation penalty received depends on each penalty amount, enforcement effectiveness for both FPS and DPS. Under DPS, it is also affected by the form of the dynamic penalty coefficient function. Under FPS, the simulation results show that the strategy probabilities of two parties fluctuate periodically around (0.38,0.83), (0.30,0.63), and (0.25,0.50) when three levels of individual penalty amount are introduced (Table 9 and Figure 1a). It means that as the penalty amount increases, the centers of the fluctuation of two parties’ strategy probabilities gradually decrease, and the probabilities of committing traffic violations and enforcing traffic rules are both unstable. Under DPS, the strategy probabilities of two parties converge to the ESSs (i.e., (0.67,1), (0.40,0.90), and (0.34,0.74)) (Table 9 and Figure 1b). It means that the probabilities of committing traffic violations and enforcing traffic rules are both significantly reduced, and their strategy probabilities will reach an equilibrium solution as the penalty amount increases. These results show that increasing penalty amount and using DPS can reduce cyclists’ probability of committing traffic violations and achieve a stable enforcement outcome as long as the traffic violation penalty is higher than the perceived relative benefit and the enforcement cost is smaller than the enforcement benefit. Such results are consistent with most of the previous studies. Kim and Kim [36] concluded that increasing the penalty amount can reduce the probability of speeding among drivers. Wong et al. [32] suggested that increasing the penalty (e.g., a higher fine and a demerit point system) for running a red light can effectively reduce such behavior among public light bus drivers. Paola et al. [33] suggested that using a demerit point system instead of a money-only system can be more effective in reducing traffic violations among drivers based on the empirical data from Italy. Such results are different from a related study by Bjørnskau and Elvik [35]. They concluded that increasing the penalty amount had no effect on the probability of speeding among drivers. A possible reason for such difference is that Bjørnskau and Elvik [35] did not consider penalties issued as a payoff for law enforcement.

Additional simulation results show that as the enforcement effectiveness (r) increases (from 0.5 to 1), law enforcement can effectively reduce the probability of traffic violations (from 52% to 34%) with fewer intersections/segments to monitor (from 79% to 45%) in the equilibrium solution under DPS (Table 9 and Figure 2). These results show the importance of the enforcement effectiveness in reducing traffic violations. Motor vehicle safety [52] reported that several measures (e.g., automated red-light enforcement and automated speed-camera enforcement) that can improve the enforcement effectiveness can reduce drivers’ traffic violations (e.g., red-light running and speeding). Additional studies are needed to evaluate the cost-benefit of reducing traffic violations through improving the enforcement effectiveness. 

To evaluate the impacts of how different forms of DPS can affect the equilibrium solution, additional simulations are conducted by introducing three types of dynamic penalty coefficient functions (i.e., k=1+p, $k=1+p^{2}$, and $k=1+2p-p^{2}$). Figure 3 reflects the relationship between probabilities of committing traffic violations and the dynamic penalty coefficient. As shown in Figure 4, the strategy probabilities of two parties converge to the ESSs (i.e., (0.34,0.45), (0.39,0.52), and (0.31,0.39)). These results illustrate that when the dynamic penalty coefficient in DPS follows $k=1+2p-p^{2}$, the probabilities of committing traffic violations and enforcing traffic rules are both the lowest. These results suggest that the effectiveness of the DPS depends on the growth rate of the dynamic penalty coefficient, and a decreasing growth rate of the dynamic penalty coefficient can yield a more effective result. It means that using a stricter penalty at the beginning and then gradually relaxing them might achieve a better enforcement outcome.

To sum up, DPS can be more effective when the traffic violation penalty is more than the perceived relative benefit and enforcement cost is less than the enforcement benefit In addition, increasing penalties (e.g., higher fines and a demerit point system), improving enforcement effectiveness (e.g., automatic speeding/red-light running/ retrograding violation detection and response system), and adopting a DPS with a decreasing growth rate in the dynamic penalty coefficient over a FPS can be more effective in reducing traffic violations.

### 4.3. The Effects of Perceived Relative Benefit

The total amount of perceived relative benefit depends on risk attitude coefficients, loss aversion coefficient, and decision weight. Under FPS, the simulation results show that the strategy probabilities of two parties fluctuate periodically (i.e., unstable) around (0.43,0.20), (0.43,0.40), and (0.43,0.60) with three levels of perceived relative benefit (Table 9 and Figure 5a). It means that as the perceived relative benefit decreases, the centers of the fluctuation of cyclists’ strategy probabilities remain unchanged and those of law enforcement’s strategy probabilities significantly decrease. Under DPS, the strategy probabilities of two parties converge to the ESSs (i.e., (0.52,0.27), (0.52,0.53), and (0.52,0.79)) (Table 9 and Figure 5b). It means that the probabilities of committing traffic violations remain unchanged, while the probabilities of enforcing traffic rules are significantly reduced as the perceived relative benefit decreases, and their strategy probabilities will reach an equilibrium solution. These results show that decreasing perceived relative benefit within a certain range (less than the traffic violation penalty) and using DPS can gain a stable result in terms of reducing cyclists’ probability of committing traffic violations compared to using FPS.

The perceived relative benefit depends on several attitude-related factors, such as risk attitude coefficients, loss aversion coefficient, and decision weight. These factors are often acquired by analyzing stated preference surveys and field observations [53,54]. In addition, we assume $\omega \left(p_{1}\right)+\omega \left(p_{2}\right)=0.9$ under the condition that the decision weights of complementary events sum to less than 1 [43].

Under DPS, the simulation results show that the strategy probabilities of two parties converge to the ESSs (i.e., (0.30,0.07), (0.30,0.23), and (0.30,0.50)) when three levels of risk attitude coefficients are introduced (Table 9 and Figure 6). These results show that as the risk attitude coefficient decreases, it takes much longer for cyclists to reach the equilibrium solution and the probability of their committing traffic violations at the equilibrium remain the same; while it takes much longer for law enforcement to reach the equilibrium solution, but the probability of their enforcing traffic rules at the equilibrium significantly reduces. These results suggest that the higher risk that cyclists perceive towards committing traffic violations, the lower probability of enforcing traffic rules is needed to maintain the same level of probability of committing traffic violations, and the longer it takes to reach the equilibrium solution. 

Under DPS, the simulation results show that the strategy probabilities of two parties converge to the ESSs (i.e., (0.31,0.50), (0.31,0.48), and (0.31,0.46)) with three levels of loss aversion coefficient (Table 9 and Figure 7). As the loss aversion coefficient increases, the probability of committing traffic violations remains the same, while the probability of enforcing traffic rules reduces slightly. These findings indicated that if cyclists are more sensitive to possible losses towards being punished for committing traffic violations, law enforcement can control the probability of committing traffic violations to the same level with a lower probability of enforcing traffic rules.

Under DPS, the simulation results show that the strategy probabilities of two parties converge to the ESSs (i.e., (0.31,0.19), (0.31,0.34), and (0.31,0.50)) given three levels of decision weight of $p_{1}$ (the probability of not getting punished for traffic violations) (Table 9 and Figure 8). It means that as the decision weight of $p_{1}$ increases, it takes fewer time for cyclists and law enforcement to reach the equilibrium solutions, while the equilibrium probability of committing traffic violations remains the same but the equilibrium probability of enforcing traffic rules significantly increases. The results suggest that when cyclists underestimate the likelihood of being punished for traffic violations, law enforcement should adopt a higher probability of enforcing traffic rules to achieve the same level of probability of committing traffic violations.

To sum up, DPS can be more effective when the traffic violation penalty is more than the perceived relative benefit, and enforcement cost is less than the enforcement benefit. In addition, various measures that can decrease the cyclists’ perceived relative benefit (e.g., school-based education, advertisements, and training programs related to traffic violations) can be effective in achieving the same level of probability of committing traffic violations with a lower probability of enforcing traffic rules. These measures should be designed to influence cyclists’ (i) risk attitudes towards committing traffic violations, (ii) sensitivity to the possible loss when punished for committing traffic violations, (iii) and perceived likelihood of being punished.

### 4.4. The Effects of Enforcement Cost

Under FPS, the simulation results show that the strategy probabilities of two parties fluctuate periodically around (0.43,0.60), (0.71,0.60), and (0.86,0.60) under three levels of enforcement cost (Table 9 and Figure 9a). It means that as the enforcement cost decreases, the centers of the fluctuation of cyclists’ strategy probability of committing traffic violations significantly decrease, and those of law enforcement’s strategy probability of enforcing traffic rules remain the same. Under DPS, the strategy probabilities of two parties converge to the ESSs (i.e., (0.52,0.79), (0.78,0.67), and (0.89,0.62)) (Table 9 and Figure 9b). 

These results show that as the enforcement cost decreases, the probability of committing traffic violations significantly reduces while the probability of enforcing traffic rules gradually increases. Possible measures for reducing enforcement cost can include adopting more cost-effective technologies, improving system operation, etc. In addition, DPS can be more effective in achieving a stable reduction in probability of committing traffic violations when the enforcement cost reduces compared to FPS if the enforcement cost is less than the enforcement benefit.

### 4.5. The Effects of Relative Public Image Cost

Under FPS, the simulation results show that the strategy probabilities of two parties fluctuate periodically around (0.43,0.60), (0.38,0.60), and (0.30,0.60) given three levels of the relative public image cost (Table 9 and Figure 10a). As the relative public image cost increases, the centers of the fluctuation of cyclists’ strategy probability of committing traffic violations gradually decrease and those of law enforcement’s strategy probability of enforcing traffic rules remains the same. Under DPS, the strategy probabilities of two parties converge to the ESSs (i.e., (0.52,0.79), (0.45,0.83), and (0.36,0.88)) (Table 9 and Figure 10b). 

This means that as the relative public image cost increases, the probability of committing traffic violations significantly reduces, while the probability of enforcing traffic rules gradually increases. These results show that increasing relative public image cost as long as it is more than the difference of the enforcement cost minus the traffic violation penalty and using DPS can reduce the probability of committing traffic violations with a slightly increasing probability of enforcing traffic rules. These results show that when the relative public image cost increases, the probability of committing traffic violations can be effectively reduced as it provides more incentives for law enforcement to enforce traffic rules. The relative public image cost is calculated by the cost of the negative public image by not punishing violations plus the benefit of the positive public image by enforcing traffic rules. Thereby, policymakers should leverage media and education campaigns to influence public opinion to foster a social environment in which the public image cost of not enforcing traffic rules plays an important role in designing penalty strategies. 

## 5. Conclusions

In this paper, we developed an evolutionary game theory framework to understand the interaction between cyclists’ traffic violations and enforcement strategies. To evaluate the proposed framework, numerical experiments were conducted to analyze the evolutionary equilibrium stability under two law enforcement penalty strategies (FPS and DPS). Based on the cost differences among the penalty amount, enforcement effectiveness, perceived relative benefit, enforcement cost, and relative public image cost, three potential situations were studied. When the perceived relative benefit is larger than the traffic violation penalty or the enforcement cost is large than the enforcement benefit (Situations 1 and 2), the equilibrium state is very similar under FPS and DPS and both strategies are unable to reduce the probability of committing traffic violations. When the perceived relative benefit of committing traffic violations is less than the traffic violation penalty and the enforcement cost is smaller than the enforcement benefit (Situation 3), the equilibrium state (i.e., a stable expected outcome of the enforcement) can only be achieved under DPS. The numerical experiments also show that the penalty amount, enforcement effectiveness, perceived relative benefit, enforcement cost, relative public image cost, and cyclists’ attitude towards risk (risk attitude coefficients, loss aversion coefficient, and decision weight) have significant impacts on their choice of strategy.

These findings can have a few policy implications that can help to reduce traffic violations among cyclists, particularly when law enforcement resources are limited. First, cyclists are more likely to commit traffic violations if their perceived relative benefit of traffic violations is larger than the traffic violation penalty, or the enforcement cost is larger than the enforcement benefit. It is important for law enforcement to not only introduce stiffer penalty (higher fines and a demerit point system) for traffic violations but also improve enforcement effectiveness (e.g., automatic speeding/red-light running/retrograding violation detection and response system) to increase the traffic violation penalty. Second, DPS can achieve a more stable reduction in probability of committing traffic violations compared to FPS. Law enforcement may consider leveraging mobile technologies (e.g., smartphone apps) to update the penalties quickly with limited infrastructural investment and help cyclists to be more informed of the penalty changes. Third, it is important to develop educational programs and media campaigns to reduce traffic violations by influencing cyclists’ risk attitude towards traffic violations, sensitivity to the possible loss when punished for traffic violations, estimated likelihood of being punished, and the cost to the public image by not enforcing traffic rules. Last but not the least, adopting more cost-effective technologies and improving system operation can potentially reduce the cost of enforcing traffic rules which lead to fewer traffic violations. 

This study has a few limitations and can be addressed through future studies. First, some of the assumptions related to the payoffs to cyclists and law enforcement can be relaxed. Additional studies are needed to consider the impacts of the potential cost of committing traffic violations (such as increased safety risk) and the potential benefits of not committing traffic violations (such as reduced safety risk). Second, cyclists of regular bikes and e-bikes are considered similar and are represented as one agent. However, regular bike cyclists and e-bike cyclists are very different in their sociodemographic and behavioral characteristics [19,21,24,28]. In many countries, these two types of cyclists are managed differently and some e-bikes are classified as motor vehicles [55]. A potential future direction can be studying the potential differences in terms of regular bike cyclists’ and e-bike cyclists’ interaction with law enforcement. Third, additional studies are needed to calibrate the proposed model using real-world data. A four-phase study has been planned to address this issue. In Phase I, a self-reported survey will be conducted to study the potential influence of the socio-demographic variables (e.g., age, gender, and education background) of cyclists on traffic violations. In Phase II, an interactive bicycling simulator study will be conducted to evaluate cyclists’ traffic violations and enforcement strategy. Detailed post-study interviews will also be conducted to identify possible additional factors affecting the interaction between cyclists’ traffic violations and enforcement strategies. In the following phase, two arterials located in Nantong University, China (one for FPS and the other one for DPS) will be used evaluate real-world interactions between cyclists’ traffic violations and enforcement strategies. In the final phase, we are planning to collaborate with law enforcement agencies in Nantong City, China to implement the enforcement strategies designed based on aforementioned studies in the city and validate the study results. Lastly, considering the differences among the road users, the proposed approach can be applied to investigate the interaction between drivers or pedestrians and law enforcement related to traffic violations.

## Figures and Tables

**Table 1:** Payoff matrix for cyclists and law enforcement.

		Law Enforcement	
		Enforce Traffic Rules	Do Not Enforce Traffic Rules
Cyclists	Commit traffic violations	$\left(\Delta V-rC_{p},rC_{p}-C_{e}\right)$	(ΔV,−ΔI)
	Do not commit traffic violations	$\left(0,-C_{e}\right)$	(0,0)

**Table 2:** Determinants and traces of the Jacobian matrix J for five potential ESSs under FPS.

Equilibrium State	$\frac{\partial f\left(p\right)}{\partial p}$	$\frac{\partial f\left(p\right)}{\partial q}$	$\frac{\partial f\left(q\right)}{\partial p}$	$\frac{\partial f\left(q\right)}{\partial q}$	det J	tr J
$E_{1}=\left(0,0\right)$	ΔV	0	0	$-C_{e}$	$-\Delta VC_{e}$	$\Delta V-C_{e}$
$E_{2}=\left(0,1\right)$	$\Delta V-rC_{p\left(f\right)}$	0	0	$C_{e}$	$\left(\Delta V-rC_{p\left(f\right)}\right)C_{e}$	$\left(\Delta V-rC_{p\left(f\right)}\right)+C_{e}$
$E_{3}=\left(1,0\right)$	−ΔV	0	0	$rC_{p\left(f\right)}+\Delta I-C_{e}$	$-\Delta V\left(rC_{p\left(f\right)}+\Delta I-C_{e}\right)$	$\left(rC_{p\left(f\right)}+\Delta I-C_{e}\right)-\Delta V$
$E_{4}=\left(1,1\right)$	$-\left(\Delta V-rC_{p\left(f\right)}\right)$	0	0	$-(rC_{p\left(f\right)}+\Delta I-C_{e})$	$\left(\Delta V-rC_{p\left(f\right)}\right)(rC_{p\left(f\right)}+\Delta I-C_{e})$	$-\left(\Delta V-rC_{p\left(f\right)}\right)-(rC_{p\left(f\right)}+\Delta I-C_{e})$
$E_{5}=\left(p^{*},q^{*}\right)$	0	$\left(p^{*}{}^{2}-p^{*}\right)rC_{p\left(f\right)}$	$\left(q^{*}-q^{*}{}^{2}\right)(rC_{p\left(f\right)}+\Delta I)$	0	$\left(p^{*}{}^{2}-p^{*}\right)\left(q^{*}{}^{2}-q^{*}\right)(rC_{p\left(f\right)}+\Delta I)rC_{p\left(f\right)}$	0

**Table 3:** Situation 1: local stability of equilibrium.

Equilibrium	Sign of det J		Sign of tr J		Result	
	FPS	DPS	FPS	DPS	FPS	DPS
$E_{1}=\left(0,0\right)$	−	−	uncertain	uncertain	Saddle	Saddle
$E_{2}=\left(0,1\right)$	+	+	+	+	Unstable	Unstable
$E_{3}=\left(1,0\right)$	−	−	uncertain	uncertain	Saddle	Saddle
$E_{4}=\left(1,1\right)$	+	+	−	−	Stable	Stable

**Table 4:** Situation 2: local stability of equilibrium.

Equilibrium	Sign of det J		Sign of tr J		Result	
	FPS	DPS	FPS	DPS	FPS	DPS
$E_{1}=\left(0,0\right)$	−	−	uncertain	uncertain	Saddle	Saddle
$E_{2}=\left(0,1\right)$	+/−	+/−	+/uncertain	+/uncertain	Unstable/Saddle	Unstable/Saddle
$E_{3}=\left(1,0\right)$	+	+	−	−	Stable	Stable
$E_{4}=\left(1,1\right)$	−/+	−/+	uncertain/+	uncertain/+	Saddle/Unstable	Saddle/Unstable

**Table 5:** Situation 3: local stability of equilibrium.

Equilibrium	Sign of det J		Sign of tr J		Result	
	FPS	DPS	FPS	DPS	FPS	DPS
$E_{1}=\left(0,0\right)$	−	−	uncertain	uncertain	Saddle	Saddle
$E_{2}=\left(0,1\right)$	−	−	uncertain	uncertain	Saddle	Saddle
$E_{3}=\left(1,0\right)$	−	−	uncertain	uncertain	Saddle	Saddle
$E_{4}=\left(1,1\right)$	−	−	uncertain	uncertain	Saddle	Saddle
$E_{5}=\left(p^{*},q^{*}\right)$	+	+	0	−	Center	Stable

**Table 6:** Determinants and traces of the Jacobian matrix J for five potential ESSs under DPS.

Equilibrium State	$\frac{\partial f\left(p\right)}{\partial p}$	$\frac{\partial f\left(p\right)}{\partial q}$	$\frac{\partial f\left(q\right)}{\partial p}$	$\frac{\partial f\left(q\right)}{\partial q}$	det J	tr J
$E_{1}=\left(0,0\right)$	ΔV	0	0	$-C_{e}$	$-\Delta VC_{e}$	$\Delta V-C_{e}$
$E_{2}=\left(0,1\right)$	$\Delta V-rC_{p\left(d\right)}$	0	0	$C_{e}$	$\left(\Delta V-rC_{p\left(d\right)}\right)C_{e}$	$\left(\Delta V-rC_{p\left(d\right)}\right)+C_{e}$
$E_{3}=\left(1,0\right)$	−ΔV	0	0	$2rC_{p\left(d\right)}+\Delta I-C_{e}$	$-\Delta V\left(2rC_{p\left(d\right)}+\Delta I-C_{e}\right)$	$\left(2rC_{p\left(d\right)}+\Delta I-C_{e}\right)-\Delta V$
$E_{4}=\left(1,1\right)$	$-\left(\Delta V-2rC_{p\left(d\right)}\right)$	0	0	$-(2rC_{p\left(d\right)}+\Delta I-C_{e})$	$\left(\Delta V-2rC_{p\left(d\right)}\right)(2rC_{p\left(d\right)}+\Delta I-C_{e})$	$-\left(\Delta V-2rC_{p\left(d\right)}\right)-(2rC_{p\left(d\right)}+\Delta I-C_{e})$
$E_{5}=\left(p^{*},q^{*}\right)$	$q^{*}rC_{p\left(d\right)}\left(3p^{*}{}^{2}-1\right)+\Delta V\left(1-2p^{*}\right)$	$\left(p^{*}{}^{3}-p^{*}\right)rC_{p\left(d\right)}$	$2p^{*}q^{*}rC_{p\left(d\right)}\left(1-q^{*}\right)-\left(q^{*}{}^{2}-q^{*}\right)(rC_{p\left(d\right)}+\Delta I)$	$\left(1-2q^{*}\right)\left(p^{*}{}^{2}rC_{p\left(d\right)}+p^{*}\left(rC_{p\left(d\right)}+\Delta I\right)-C_{e}\right)$	$\left(q^{*}rC_{p\left(d\right)}\left(3p^{*}{}^{2}-1\right)+\Delta V\left(1-2p^{*}\right)\right)\left(1-2q^{*}\right)\left(p^{*}{}^{2}rC_{p\left(d\right)}+p^{*}\left(rC_{p\left(d\right)}+\Delta I\right)-C_{e}\right)-\left(2p^{*}q^{*}rC_{p\left(d\right)}\left(1-q^{*}\right)-\left(q^{*}{}^{2}-q^{*}\right)(rC_{p\left(d\right)}+\Delta I)\right)\left(p^{*}{}^{3}-p^{*}\right)rC_{p\left(d\right)}$	$q^{*}rC_{p\left(d\right)}\left(3p^{*}{}^{2}-1\right)+\Delta V\left(1-2p^{*}\right)+\left(1-2q^{*}\right)\left(p^{*}{}^{2}rC_{p\left(d\right)}+p^{*}\left(rC_{p\left(d\right)}+\Delta I\right)-C_{e}\right)$

**Table 7:** Values of each factor in the proposed models in Situations 1, 2, and 3.

Factor	Situation	$C_{p\left(f\right)}/C_{p\left(d\right)}$	ΔV	$C_{e}$	ΔI	r	E	α, β	λ	$\omega \left(p_{1}\right)$
$C_{p\left(f\right)}/C_{p\left(d\right)}$	Situation 1	-	50	30	20	1	-	-	-	-
	Situation 2	-	50	65	20	1	-	-	-	-
	Situation 3	-	50	30	20	1	-	-	-	-
ΔV	Situation 1	30/15	-	30	20	1	-	-	-	-
	Situation 2	30/15	-	60	20	1	-	-	-	-
	Situation 3	50/25	-	30	20	1	-	-	-	-
$C_{e}$	Situation 1	50/25	60	-	20	1	-	-	-	-
	Situation 2	20/10	30	-	10	1	-	-	-	-
	Situation 3	50/25	30	-	20	1	-	-	-	-
ΔI	Situation 1	50/25	60	30	-	1	-	-	-	-
	Situation 2	20/10	30	65	-	1	-	-	-	-
	Situation 3	50/25	30	30	-	1	-	-	-	-
r	Situation 3	-/50	30	30	20	-	-	-	-	-
k	Situation 3	-/50	30	30	20	1	-	-	-	-
α, β	Situation 3	-/60	-	30	20	1	50	-	1	0.8
λ	Situation 3	-/60	-	30	20	1	50	1	-	0.8
$\omega \left(p_{1}\right)$	Situation 3	-/60	-	30	20	1	50	1	1	-

**Table 8:** Converging speed changes of the probabilities of traffic violations and enforcing traffic rules in Situations 1 and 2.

Factor	Converging to Committing Traffic Violations (Cyclists)				Converging to Enforcing Traffic Rules (Law Enforcement)			
	Situation 1 p=1		Situation 2 p=1		Situation 1 q=1		Situation 2 q=0	
	FPS	DPS	FPS	DPS	FPS	DPS	FPS	DPS
Penalty amount increases $C_{p\left(f\right)}=$ (20 ^1^, 30 ^1^, 40 ^1^) $C_{p\left(d\right)}=\;$(10 ^1^, 15 ^1^, 20 ^1^)	Decreases (24 ^2^, 37 ^2^, 73 ^2^)	Decreases (23 ^2^, 36 ^2^, 67 ^2^)	Decreases (19 ^2^, 20 ^2^, 21 ^2^)	Decreases (18 ^2^, 19 ^2^, 20 ^2^)	Increases (97 ^2^, 51 ^2^, 36 ^2^)	Increases (98 ^2^, 52 ^2^, 36 ^2^)	Decreases (34 ^2^, 56 ^2^, 161 ^2^)	Decreases (34 ^2^, 54 ^2^, 156 ^2^)
Perceived relative benefit increases ΔV= (50 ^1^, 60 ^1^, 80 ^1^)	Increases (35 ^2^, 25 ^2^, 15 ^2^)	Increases (34 ^2^, 24 ^2^, 15 ^2^)	Increases (20 ^2^, 16 ^2^, 12 ^2^)	Increases (19 ^2^, 15 ^2^, 12 ^2^)	Increases (50 ^2^, 49 ^2^, 47 ^2^)	Increases (51 ^2^, 50 ^2^, 47 ^2^)	Decreases (83 ^2^, 85 ^2^, 86 ^2^)	Decreases (81 ^2^, 83 ^2^, 85 ^2^)
Enforcement cost increases $C_{e}=$ (40 ^1^, 50 ^1^, 60 ^1^)	Increases (67 ^2^, 56 ^2^, 27 ^2^)	Increases (62 ^2^, 50 ^2^, 25 ^2^)	Increases (33 ^2^, 31 ^2^, 30 ^2^)	Increases (32 ^2^, 30 ^2^, 29 ^2^)	Decreases (36 ^2^, 52 ^2^, 102 ^2^)	Decreases (36 ^2^, 53 ^2^, 106 ^2^)	Increases (82 ^2^, 41 ^2^, 28 ^2^)	Increases (81 ^2^, 40 ^2^, 27 ^2^)
Relative public image cost increases ΔI= (10 ^1^, 20 ^1^, 40 ^1^)	Decreases (69 ^2^, 74 ^2^, 78 ^2^)	Decreases (63 ^2^, 69 ^2^, 72 ^2^)	Decreases (30 ^2^, 31 ^2^, 33 ^2^)	Decreases (29 ^2^, 30 ^2^, 32 ^2^)	Increases (35 ^2^, 28 ^2^, 20 ^2^)	Increases (36 ^2^, 29 ^2^, 20 ^2^)	Decreases (23 ^2^, 32 ^2^, 149 ^2^)	Decreases (23 ^2^, 31 ^2^, 148 ^2^)

^1^ Value changes to factors; ^2^ The amount of time it takes to converge.

**Table 9:** Probabilities at the equilibrium and stabilities under FPS or DPS in Situation 3.

Factor	Probability and Stability of Committing Traffic Violations				Probability and Stability of Enforcing Traffic Rules			
	FPS		DPS		FPS		DPS	
	Probability	Stability	Probability	Stability	Probability	Stability	Probability	Stability
Penalty amount increases	0.38	Unstable	0.67	70 ^1^	0.83	Unstable	1	70 ^1^
	0.30		0.40	90 ^1^	0.63		0.90	90 ^1^
	0.25		0.34	110 ^1^	0.50		0.74	110 ^1^
Enforcement effectiveness increases	-	-	0.52	150 ^1^	-	-	0.79	150 ^1^
	-	-	0.43	150 ^1^	-	-	0.60	150 ^1^
	-	-	0.34	150 ^1^	-		0.45	150 ^1^
Changes in growth rate of dynamic penalty coefficient	-	-	0.34	140 ^1^	-	-	0.45	140 ^1^
	-	-	0.39	140 ^1^	-	-	0.52	140 ^1^
	-	-	0.31	140 ^1^	-	-	0.39	140 ^1^
Perceived relative benefit increases	0.43	Unstable	0.52	500 ^1^	0.20	Unstable	0.27	500 ^1^
	0.43		0.52	200 ^1^	0.40		0.53	200 ^1^
	0.43		0.52	170 ^1^	0.60		0.79	170 ^1^
Risk attitude coefficients increase	-	-	0.30	1000 ^1^	-	-	0.07	1000 ^1^
	-	-	0.30	300 ^1^	-	-	0.23	300 ^1^
	-	-	0.30	150 ^1^	-	-	0.50	150 ^1^
Loss aversion coefficient increases	-	-	0.31	150 ^1^	-	-	0.50	150 ^1^
	-	-	0.31	150 ^1^	-	-	0.48	150 ^1^
	-	-	0.31	150 ^1^	-	-	0.46	150 ^1^
Decision weight increases	-	-	0.31	300 ^1^	-	-	0.19	300 ^1^
	-	-	0.31	200 ^1^	-	-	0.34	200 ^1^
	-	-	0.31	140 ^1^	-	-	0.50	140 ^1^
Enforcement cost increases	0.43	Unstable	0.52	150 ^1^	0.60	Unstable	0.79	150 ^1^
	0.71		0.78	300 ^1^	0.60		0.67	300 ^1^
	0.86		0.89	500 ^1^	0.60		0.62	500 ^1^
Relative public image cost increases	0.43	Unstable	0.52	150 ^1^	0.60	Unstable	0.79	150 ^1^
	0.38		0.45	150 ^1^	0.60		0.83	150 ^1^
	0.30		0.36	150 ^1^	0.60		0.88	150 ^1^

^1^ Time takes to reach equilibrium solution.

## Appendix B. First-oRder Partial Derivatives of $p^{*}$, $q^{*}$ and ΔV

(A1) $$\frac{\partial p^{*}}{\partial C_{p\left(d\right)}}=\frac{\Delta I\sqrt{r^{2}C_{p\left(d\right)}{}^{2}+r\left(2C_{p\left(d\right)}\Delta I+4C_{p\left(d\right)}C_{e}\right)+\Delta I^{2}}-r\left(C_{p\left(d\right)}\Delta I+2C_{p\left(d\right)}C_{e}\right)-\Delta I^{2}}{2rC_{p\left(d\right)}{}^{2}\sqrt{r^{2}C_{p\left(d\right)}{}^{2}+r\left(2C_{p\left(d\right)}\Delta I+4C_{p\left(d\right)}C_{e}\right)+\Delta I^{2}}}<0$$

(A2) $$\frac{\partial q^{*}}{\partial C_{p\left(d\right)}}=\frac{\Delta Vr\sqrt{r^{2}C_{p\left(d\right)}{}^{2}+r\left(2C_{p\left(d\right)}\Delta I+4C_{p\left(d\right)}C_{e}\right)+\Delta I^{2}}+\Delta Vr^{2}C_{p\left(d\right)}+\left(2C_{e}+\Delta I\right)\Delta Vr}{\left(r^{2}C_{p\left(d\right)}{}^{2}+2rC_{p\left(d\right)}C_{e}+\Delta I^{2}\right)\sqrt{r^{2}C_{p\left(d\right)}{}^{2}+r\left(2C_{p\left(d\right)}\Delta I+4C_{p\left(d\right)}C_{e}\right)+\Delta I^{2}}+r^{3}C_{p\left(d\right)}{}^{3}+r^{2}\left(C_{p\left(d\right)}{}^{2}\Delta I+4C_{p\left(d\right)}{}^{2}C_{e}\right)-r\left(C_{p\left(d\right)}\Delta I^{2}+4C_{p\left(d\right)}C_{e}\Delta I\right)-\Delta I^{3}}<0$$

(A3) $$\frac{\partial p^{*}}{\partial r}=\frac{\Delta I\sqrt{r^{2}C_{p\left(d\right)}{}^{2}+r\left(2C_{p\left(d\right)}\Delta I+4C_{p\left(d\right)}C_{e}\right)+\Delta I^{2}}-r\left(C_{p\left(d\right)}\Delta I+2C_{p\left(d\right)}C_{e}\right)-\Delta I^{2}}{2r^{2}C_{p\left(d\right)}\sqrt{r^{2}C_{p\left(d\right)}{}^{2}+r\left(2C_{p\left(d\right)}\Delta I+4C_{p\left(d\right)}C_{e}\right)+\Delta I^{2}}}<0$$

(A4) $$\frac{\partial q^{*}}{\partial r}=\frac{\Delta VC_{p\left(d\right)}\sqrt{r^{2}C_{p\left(d\right)}{}^{2}+r\left(2C_{p\left(d\right)}\Delta I+4C_{p\left(d\right)}C_{e}\right)+\Delta I^{2}}+\Delta VrC_{p\left(d\right)}{}^{2}+\left(2C_{e}+\Delta I\right)\Delta VC_{p\left(d\right)}}{\left(r^{2}C_{p\left(d\right)}{}^{2}+2rC_{p\left(d\right)}C_{e}+\Delta I^{2}\right)\sqrt{r^{2}C_{p\left(d\right)}{}^{2}+r\left(2C_{p\left(d\right)}\Delta I+4C_{p\left(d\right)}C_{e}\right)+\Delta I^{2}}+r^{3}C_{p\left(d\right)}{}^{3}+r^{2}\left(C_{p\left(d\right)}{}^{2}\Delta I+4C_{p\left(d\right)}{}^{2}C_{e}\right)-r\left(C_{p\left(d\right)}\Delta I^{2}+4C_{p\left(d\right)}C_{e}\Delta I\right)-\Delta I^{3}}<0$$

(A5) $$\frac{\partial q^{*}}{\partial \Delta V}=\frac{2}{\sqrt{r^{2}C_{p\left(d\right)}{}^{2}+r\left(2C_{p\left(d\right)}\Delta I+4C_{p\left(d\right)}C_{e}\right)+\Delta I^{2}}+rC_{p\left(d\right)}-\Delta I}>0$$

(A6) $$\frac{\partial p^{*}}{\partial C_{e}}=\frac{1}{\sqrt{r^{2}C_{p\left(d\right)}{}^{2}+r\left(2C_{p\left(d\right)}\Delta I+4C_{p\left(d\right)}C_{e}\right)+\Delta I^{2}}}>0$$

(A7) $$\frac{\partial q^{*}}{\partial C_{e}}=\frac{2\Delta VrC_{p\left(d\right)}}{\left(r^{2}C_{p\left(d\right)}{}^{2}+2rC_{p\left(d\right)}C_{e}+\Delta I^{2}\right)\sqrt{r^{2}C_{p\left(d\right)}{}^{2}+r\left(2C_{p\left(d\right)}\Delta I+4C_{p\left(d\right)}C_{e}\right)+\Delta I^{2}}+r^{3}C_{p\left(d\right)}{}^{3}+r^{2}\left(C_{p\left(d\right)}{}^{2}\Delta I+4C_{p\left(d\right)}{}^{2}C_{e}\right)-r\left(C_{p\left(d\right)}\Delta I^{2}+4C_{p\left(d\right)}C_{e}\Delta I\right)-\Delta I^{3}}<0$$

(A8) $$\frac{\partial p^{*}}{\partial \Delta I}=\frac{\sqrt{r^{2}C_{p\left(d\right)}{}^{2}+r\left(2C_{p\left(d\right)}\Delta I+4C_{p\left(d\right)}C_{e}\right)+\Delta I^{2}}-rC_{p\left(d\right)}-\Delta I}{2rC_{p\left(d\right)}\sqrt{r^{2}C_{p\left(d\right)}{}^{2}+r\left(2C_{p\left(d\right)}\Delta I+4C_{p\left(d\right)}C_{e}\right)+\Delta I^{2}}}<0$$

(A9) $$\frac{\partial q^{*}}{\partial \Delta I}=\frac{\Delta V\sqrt{r^{2}C_{p\left(d\right)}{}^{2}+r\left(2C_{p\left(d\right)}\Delta I+4C_{p\left(d\right)}C_{e}\right)+\Delta I^{2}}-\Delta VrC_{p\left(d\right)}-\Delta V\Delta I}{\left(r^{2}C_{p\left(d\right)}{}^{2}+2rC_{p\left(d\right)}C_{e}+\Delta I^{2}\right)\sqrt{r^{2}C_{p\left(d\right)}{}^{2}+r\left(2C_{p\left(d\right)}\Delta I+4C_{p\left(d\right)}C_{e}\right)+\Delta I^{2}}+r^{3}C_{p\left(d\right)}{}^{3}+r^{2}\left(C_{p\left(d\right)}{}^{2}\Delta I+4C_{p\left(d\right)}{}^{2}C_{e}\right)-r\left(C_{p\left(d\right)}\Delta I^{2}+4C_{p\left(d\right)}C_{e}\Delta I\right)-\Delta I^{3}}>0$$

(A10) $$\frac{\partial \Delta V}{\partial \alpha }=E^{\alpha }\omega \left(p_{1}\right)logE-\lambda \omega \left(p_{2}\right){\left(C_{p\left(d\right)}-E\right)}^{\alpha }log\left(C_{p\left(d\right)}-E\right)>0$$

(A11) $$\frac{\partial \Delta V}{\partial \lambda }=-\omega \left(p_{2}\right){\left(C_{p\left(d\right)}-E\right)}^{\alpha }<0$$

(A12) $$\frac{\partial \Delta V}{\partial \omega \left(p_{1}\right)}=E^{\alpha }>0$$
